# Measurement System for Short-Pulsed Magnetic Fields

**DOI:** 10.3390/s23031435

**Published:** 2023-01-28

**Authors:** Voitech Stankevič, Skirmantas Keršulis, Justas Dilys, Vytautas Bleizgys, Mindaugas Viliūnas, Vilius Vertelis, Andrius Maneikis, Vakaris Rudokas, Valentina Plaušinaitienė, Nerija Žurauskienė

**Affiliations:** 1 Department of Functional Materials and Electronics, Center for Physical Sciences and Technology, Sauletekio Ave. 3, 10257 Vilnius, Lithuania; 2 Faculty of Electronics, Vilnius Gediminas Technical University, 10223 Vilnius, Lithuania; 3 Institute of Chemical Physics, Faculty of Physics, Vilnius University, 03225 Vilnius, Lithuania; 4 Faculty of Chemistry and Geosciences, Vilnius University, 03225 Vilnius, Lithuania

**Keywords:** colossal magnetoresistance, MOCVD technology, nanostructured manganite films, resistance relaxation processes, pulsed magnetic field, magnetic field sensors, magnetic field measurement system

## Abstract

A measurement system based on the colossal magnetoresistance CMR-B-scalar sensor was developed for the measurement of short-duration high-amplitude magnetic fields. The system consists of a magnetic field sensor made from thin nanostructured manganite film with minimized memory effect, and a magnetic field recording module. The memory effect of the La_1−x_Sr_x_(Mn_1−y_Co_y_)_z_O_3_ manganite films doped with different amounts of Co and Mn was investigated by measuring the magnetoresistance (MR) and resistance relaxation in pulsed magnetic fields up to 20 T in the temperature range of 80–365 K. It was found that for low-temperature applications, films doped with Co (LSMCO) are preferable due to the minimized magnetic memory effect at these temperatures, compared with LSMO films without Co. For applications at temperatures higher than room temperature, nanostructured manganite LSMO films with increased Mn content above the stoichiometric level have to be used. These films do not exhibit magnetic memory effects and have higher MR values. To avoid parasitic signal due to electromotive forces appearing in the transmission line of the sensor during measurement of short-pulsed magnetic fields, a bipolar-pulsed voltage supply for the sensor was used. For signal recording, a measurement module consisting of a pulsed voltage generator with a frequency up to 12.5 MHz, a 16-bit ADC with a sampling rate of 25 MHz, and a microprocessor was proposed. The circuit of the measurement module was shielded against low- and high-frequency electromagnetic noise, and the recorded signal was transmitted to a personal computer using a fiber optic link. The system was tested using magnetic field generators, generating magnetic fields with pulse durations ranging from 3 to 20 μs. The developed magnetic field measurement system can be used for the measurement of high-pulsed magnetic fields with pulse durations in the order of microseconds in different fields of science and industry.

## 1. Introduction

In recent years, pulsed magnetic fields have been widely used in different areas of science and industry. For the measurement of these magnetic fields, several techniques, which depend on magnetic field strength, homogeneity, and variation in time, as well as the required accuracy, have been used.

The available measurement techniques in pulsed magnetic field technology can be categorized into two types. The first one obtains the signal proportional to the time derivative of magnetic fields, *dB*/*dt*. The advantage of this method is the high voltage at the sensor’s output, as the conventional pulsed field has a high *dB*/*dt* (∼10,000 T/s) due to the short pulse duration and high magnetic field amplitude. Although it is the oldest method (B-dot sensor, induction coil), nowadays it is widely used in such applications as plasma science (RFX-mod2 toroidal machine) [1], for particle accelerator magnets, coilgun and railgun systems, and other fields [2,3]. Another type of sensor that also operates on practically the same principle is the fluxgate sensor. Recently, great progress has been made in the accuracy of measurements of this type of sensor and in the improvement of the signal-to-noise ratio [4]. Depending on the area of application, such sensors can be large in size (about 50 mm for detecting cardiac activity [5]) as well as miniaturized. The miniaturized sensors are manufactured with PCB technology, allowing the construction of sensors measuring several tens of millimeters [6]. However, as the measured signal in this case is proportional to the derivative of the magnetic pulse, to obtain the real magnetic pulse shape, the signal has to be integrated. This leads to error accumulation throughout the integration period [7]. Other measurement techniques are based on the Hall effect [8], and several magnetoresistivity effects such as anisotropic magnetoresistance (AMR), giant magnetoresistance (GMR), and tunneling magnetoresistance (TMR) [9,10,11]. The output signal of these sensors directly represents the magnitude of the magnetic field; however, large *dB*/*dt* in this case is a disadvantage, because it induces pick-up noise, and it becomes difficult to extract the relevant experimental data. However, these types of sensors are most often used to measure weak magnetic fields, because the saturation field is below the milli-tesla range [11,12,13]. Moreover, all these methods have one common disadvantage: they are sensitive to the direction of the magnetic field. This means that the magnetic field direction should be known in advance and it should not change during the measurement. This problem can be solved by using three orthogonally positioned sensors [4,14], which allow the measurement of not only the magnitude, but also the direction of the magnetic field. This is especially essential in certain industrial, medical, robotic, or virtual reality applications. A three-dimensional magnetic field sensor based on a single spin–orbit torque device [15], a three-dimensional magnetometer based on a simple Hall device in a planar geometry [14,16], or a three-axis induction sensor [17] can be used for this purpose. However, such a solution is good for measuring relatively long-pulsed magnetic fields. For measurement of short pulses, the increased number of wires to power the sensors together with the effect of induced voltage in these wires caused by the electromotive force strongly increases the measurement error. Moreover, the use of 3D design makes the set-ups bulky with relatively large dimensions, which causes problems when measuring locally, or in very small volumes. Meanwhile, for many pulsed field applications, the basic parameter is the magnitude of the magnetic field. The direction could be defined by using an additional anisotropic sensor. 

Recently, it was demonstrated that high-amplitude pulsed magnetic fields could be measured using novel CMR-B-scalar sensors based on the colossal magnetoresistance effect (CMR) of polycrystalline nanostructured manganite (La-Sr-Mn-O) films [18,19,20]. These sensors can measure high-magnitude pulsed magnetic fields of millisecond duration in very small volumes. It was concluded in [19] that differential B-dot sensors which have a directional response can be replaced by CMR sensors which are viable for railgun applications where directionality is not required. These sensors have the advantage of measuring the magnetic field independently of the direction, which allows for their easier installation into the measuring position because it does not require an exact orientation of the sensor concerning the magnetic field direction. CMR-B-scalar sensors have been used to measure the magnetic field distribution and magnetic diffusion processes in railguns [18,19], the distribution of highly inhomogeneous transient magnetic fields during coilgun experiments, and the magnetic fields of nondestructive dual-coil pulsed-field magnets up to a megagauss [20]. However, all of these sensors have been used to measure long-pulsed magnetic fields (usually more than 1 ms) [18,20,21]. At such pulse durations, the memory effect and the induced voltage in the signal transmission line only slightly affect the measurement accuracy.

Applications in plasma science, condensed matter physics [22,23], magnetic flux compression [24,25], or magnetic pulse welding (MPW) [26,27] require sensors measuring high-pulsed magnetic fields with a pulse duration of about several microseconds. The basic requirements for fast sensors are the absence of magnetic memory effects, and high accuracy and temporal resolution. The memory effects in manganite-based CMR-sensors are related to the magnetization relaxation of the manganite films after the exposure to an external magnetic field. Due to the close relationship between transport properties (conductivity) and magnetization of manganite material, the investigation of magnetoresistance relaxation provides an indirect method for the study of the magnetic relaxation processes in these films [28]. Our previous results demonstrated that the resistance relaxation of manganites strongly depends on the ambient temperature [29] and is governed by several different magnetic relaxation mechanisms. Moreover, it was shown [30] that the electrical transport and magnetic properties of nanostructured films strongly depend on the partial replacement of manganese atoms with cobalt ones. It was found that a remnant resistivity, after switching off the magnetic field, decreases with the increase in Co content. Therefore, to minimize the magnetic memory effects, films doped with higher amount of cobalt (Co/(La + Sr) 1 ≥ 0.12) are preferable for fast magnetic sensor applications at cryogenic temperatures due to their higher sensitivity and lower remnant resistivity change in comparison with LSMO films [31]. 

Another important parameter for magnetic field sensors is the magnetoresistance magnitude at different temperatures. It was shown that the increase in Mn excess, i.e., the increase in the ratio Mn/(La + Sr) > 1, shifts the insulator-metal transition temperature *T*_m_ to the higher-temperature region and, as a result, leads to higher magnetoresistance values at temperatures higher than 320 K [32]. For this reason, one of the goals of this work was to study the possibility of using manganite films to measure short-pulsed magnetic fields at low and high temperatures. For these purposes, films of manganites doped with Co and films with Mn excess were selected.

One more problem that needs to be solved to measure short-pulsed magnetic fields is the high electromotive force (EMF) signal appearing in the transmission line of the sensor due to the rapidly changing magnetic field. A twisted pair cable or an additional compensation loop [33] can partially solve this problem. Moreover, the EMF contribution can be subtracted out by pulsing the field in positive and negative directions and then processing the data [34]. However, this method is impossible when the experiments cannot be repeated under the same conditions. Other methods to subtract the EMF include using an AC supply for the sensor. For example, a 10 kHz AC was used to modulate the supply of the Hall sensor for correction of the output voltage [35]. However, for the measurement of magnetic field pulses with high amplitudes and microsecond durations, this method becomes complicated due to the limitation of temporal resolution and problem of synchronization during signal recording when voltage has a sinusoidal shape. 

In this study, we present the results of the investigation of the magnetoresistance and the resistance relaxation of La_1−x_Sr_x_(Mn_1−y_Co_y_)_z_O_3_ films, which can be used for the fabrication of magnetic sensors. A magnetic field meter, which is capable of measuring high-pulsed magnetic fields with a pulse duration of several microseconds, is proposed.

## 2. Materials and Methods 

### 2.1. Film Preparation 

A series of La_1−x_Sr_x_(Mn_1−y_Co_y_)_z_O_3_ films with a thickness of 350 nm were deposited using a Pulsed-Injection Metal-Organic Chemical Vapor Deposition (PI MOCVD) technique onto a polycrystalline Al_2_O_3_ substrate. An organic solution containing the mixture of metal-organic precursors (La(thd)_3_, Sr(thd)_2_, Mn(thd)_3_, and Co(thd)_3_ (thd is 2,2,6,6-tetramethyl- 3,5-heptandionate)) dissolved in the monoglyme was used as the source of film elements. During the growing process, using the injector, microdoses of this organic solution were injected into the diffuser and heated up to the temperature of about 300 °C. Inside of the diffuser after flash evaporation, the resulting vapor mixture was transported by Ar + O_2_ (3:1) gas towards the heated substrate positioned in the hot wall reactor. Growing of the film was performed at a temperature of 750 °C. After the completion of the growth process, the film was post-annealed for 10 min in oxygen in the same reactor to ensure the required oxygen saturation level in the films. The ratio of the metal organic precursors was varied to obtain the desired chemical composition of the film. A detailed description of the film growth technics and processes is presented in [36,37,38]. 

### 2.2. Characterization 

**Figure 1:**
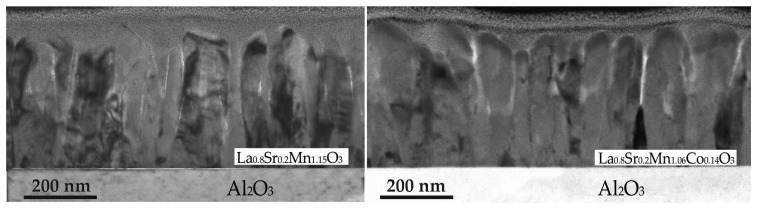
TEM images of the film with different chemical composition.

The elemental composition of the films was determined using inductively coupled plasma high-resolution mass spectrometry (ICP-MS) with a resolution of ± 0.01. The microstructure of the films was investigated by Transmission Electron Microscopy (TEM). The low-magnification cross-sectional TEM images of the films with different compositions are shown in Figure 1. As can be seen, the films consist of crystallite columns, which are spread throughout the whole film thickness with their long axis arranged perpendicular to the substrate. However, no fundamental difference between the films with different chemical compositions can be observed. The study of the grown film structure by XRD (not presented) also showed only the characteristic peaks associated with the Al_2_O_3_ substrate and polycrystalline LSMO or LSMCO films with a perovskite-like crystal structure. 

For the electric transport and magnetoresistance (MR) measurements, two Ag electrodes with a Cr sublayer were thermally deposited and post-annealed at 450 °C for 1 h in Ar atmosphere. 

The resistivity *ρ* dependence on temperature was investigated in a closed-cycle helium gas cryocooler in the temperature range of 77–310 K and in a liquid thermostat in the range of 290–365 K. The MR was defined as MR = [*ρ*(*B*)/*ρ*(0) − 1] × 100%, where *ρ*(*B*) and *ρ*(0) correspond to the resistivity in the magnetic field and without it, respectively. The MR measurements were performed in the temperature range of 80–365 K by applying magnetic field pulses with durations of 1 ms and amplitudes up to 20 T, generated by a non-destructive pulsed magnet based on a capacitor bank discharged through a multi-shot magnetic field coil [39]. To measure the resistance relaxation after the switch-off of the magnetic field pulse, a special coil with a non-metallic outer casing made from polyamide material was fabricated. This allowed the avoidance of a lagging “tail” of the magnetic field pulse after the current was switched off [29]. Using this coil it was possible to generate 200 μs duration half-sine-waveform magnetic field pulses with amplitudes of up to 10 T. The response signal *V*_res_ was recorded across the sample by passing a current of 0.2 mA through it. Two types of pulsed short-duration magnetic field generators were used for testing the created magnetic field measurement system. The design of these generators is described below.

## 3. Results and Discussion

### 3.1. Resistivity of Nanostructured LSMO Films: Dependence on Composition and Ambient Temperature

**Figure 2:**
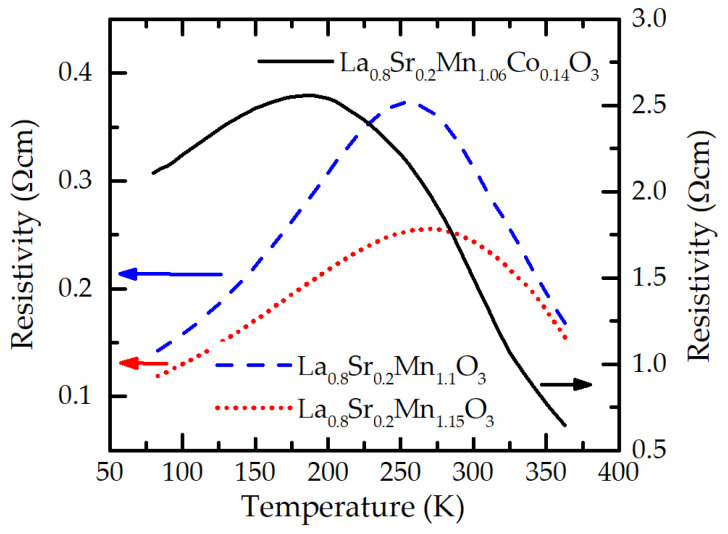
Resistivity vs. temperature dependences of LSMO and LSMCO films with different Mn and Co contents.

The resistivity vs. temperature dependences for Co-doped La_0.8_Sr_0.2_Mn_1.06_Co_0.14_O_3_ (LSMCO) films as well as films not containing Co, La_0.8_Sr_0.2_Mn_1.1_O_3_ (LSM(1.10)O) and La_0.8_Sr_0.2_Mn_1.15_O_3_ (LSM(1.15)O) are presented in Figure 2. One can see that films with Co content have the resistivity maximum (*ρ*_m_) of 2.5 Ωcm at the insulator–metal transition temperature *T*_m_ = 180 K, while the *T*_m_ of films without Co is shifted to higher temperatures: 250 K and 275 K for films with Mn/(La + Sr) = 1.1 and 1.15, respectively. At the same time, the resistivity maximum of these films is decreased: 0.36 Ωcm and 0.25 Ωcm, respectively.

**Figure 3:**
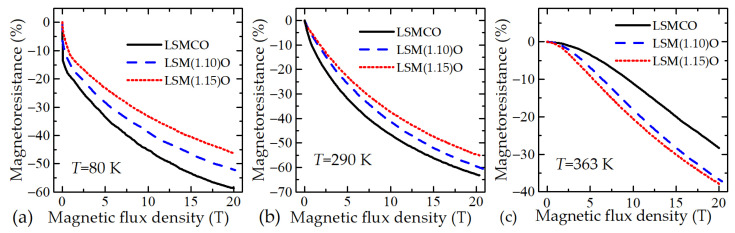
MR dependences on magnetic flux density for films with different Mn and Co contents at various ambient temperatures: (**a**) 80 K, (**b**) 290 K, (**c**) 363 K.

The dependences of MR on the magnetic flux density *B* of the LSMCO and LSMO films with various doping levels at three different temperatures are shown in Figure 3. It is evident that at 80 K and 290 K (see Figure 3a,b, respectively) the films doped with Co have the highest MR magnitude when compared with the films without it. A comparison of the magnetoresistance of the LSMO films (without Co) shows that at these temperatures the films with the amount of Mn excess equal to 1.1 have the highest MR. However, the increase in the temperature up to 363 K (Figure 3c) causes an opposite relationship. The LSMO film with Mn excess of 1.15 has the highest MR value. This is caused by the temperature of maximum resistance of this film shifting towards the higher temperatures (see Figure 2). Analysis of the obtained results allows us to conclude that the films with Co doping are more useful for the production of magnetic field sensors operating at low temperatures; however, for measuring the magnetic fields at high temperatures (higher than room temperature), the LSMO films with an increased amount of Mn without Co doping are preferable.

**Figure 4:**
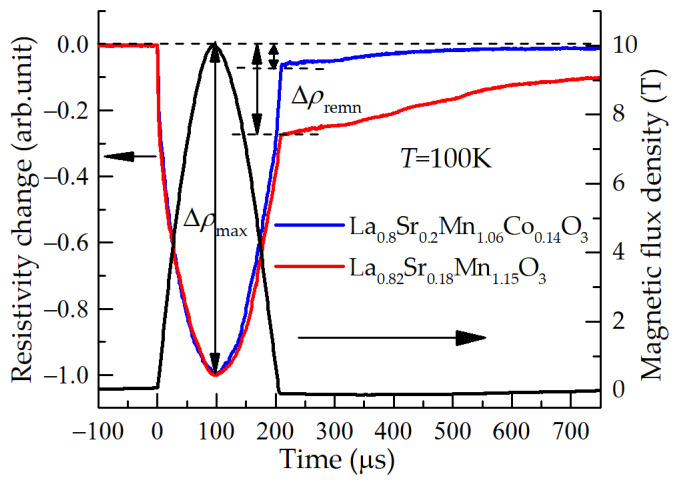
Magnetic field pulse (right scale) and resistivity change of the films with different chemical composition (left scale) during and after this pulse.

Another important parameter of the magnetic sensor is its memory effect and the resistance relaxation after the magnetic field is switched off. This parameter is responsible for the sensor’s ability to measure rapid field changes and short pulses of magnetic fields. Figure 4 presents the magnetic field pulse and resistivity change of two different films during and after the magnetic pulse, measured at a temperature of 100 K. To compare these two different films, the change of the resistivity after the magnetic pulse was switched off (at time instant *t* = 200 µs) was normalized to its maximal change at maximal magnetic field value (at *t* = 100 µs), and was expressed as:

(*ρ*(*B*_switch-off_) − *ρ*(0))/(*ρ*(0) − *ρ*(*B*_max_)) = (Δ*ρ*_remn_)/(Δ*ρ*_max_) (1)

One can see that when magnetic field drops to zero, the resistance of the films does not return to its initial value, but to some remnant one and then relaxes over hundreds of microseconds. 

**Figure 5:**
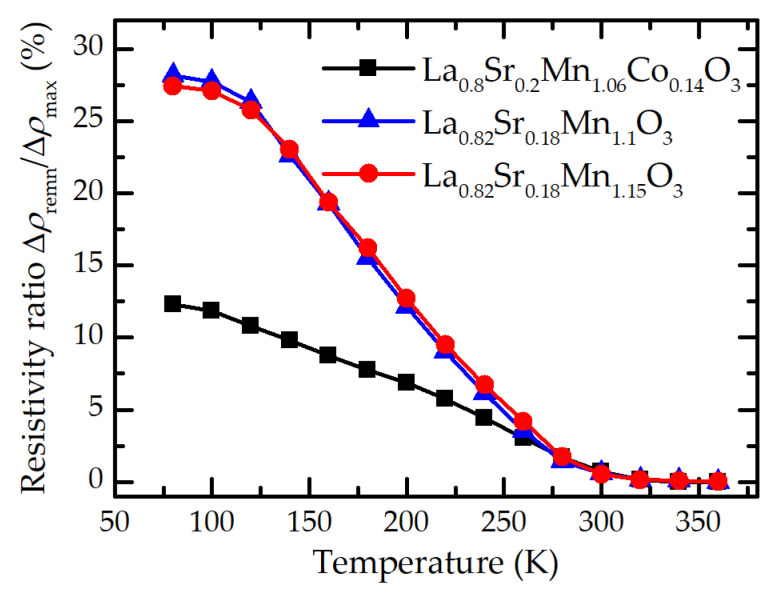
Absolute value of remnant resistivity normalized to the maximal resistivity change (at *B* = 10 T).

The values of remnant resistivity and relaxation processes of the films with different chemical compositions are presented in Figure 5. It is evident that doping these films with Co caused a decrease in normalized remnant resistivity. Moreover, remnant resistivity of these films also decreased with the increase in temperature, and at room or higher temperatures the memory effect disappears. It could be explained by a transition of the films from a ferromagnetic to a paramagnetic state, therefore, after the switch-off of the magnetic field the film returns to its initial resistance value. More detailed analysis of the relaxation process of the films with different Co composition was presented in the study [31]. An analogous dependence of the remnant resistivity on temperature is also observed in the films without Co doping. However, at low temperatures, this magnitude is almost twice as high as that for Co-doped films. Nevertheless, at high temperatures this magnetic memory effect vanishes. 

Based on the obtained results, we can conclude that for the application of manganite films as magnetic field sensors which are capable of measuring short-pulsed fields at low temperatures, the Co-doped films are preferable. For room or higher temperature applications, it is worthwhile to use Co-free LSMO films with excess amounts of Mn. 

### 3.2. Magnetic Field Sensor

**Figure 6:**
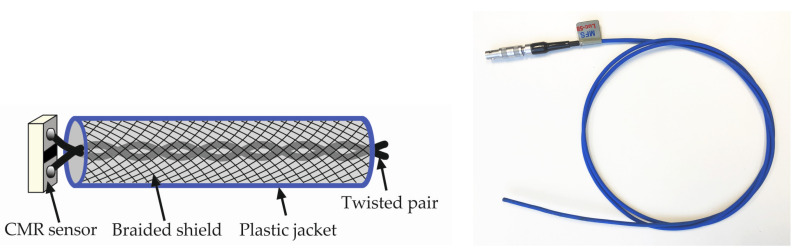
The cross-section (**left**) and picture of the sensor (**right**).

Based on the obtained results, the LSMO films with Mn content of 1.15 and not containing Co were used for fabrication of the sensor operating at room temperature and above. After the deposition of La_0.82_Sr_0.18_Mn_1.15_O_3_ film on a polycrystalline Al_2_O_3_ substrate by using a photo lithography and chemical etching, the area of the film was reduced to 0.4 × 0.2 mm^2^. Then, chromium and silver layers were thermally evaporated in a way in which silver partly overlapped with the LSMO layer. Thus, an electric contact was created and the remaining part of the LSMO film defined the active volume of 400 × 50 × 0.40 μm^3^. Next, bifilarly twisted wires were soldered perpendicular to the surface of the film. The active area was then covered by hot-melt adhesive material in order to protect the film from atmospheric conditions and to strengthen the solder joints. The twisted wire cable was additionally shielded against high-frequency noise using a braided sleeve and covered with a flexible plastic tube. Finally, a connector was attached to the cable. The cross-section and picture of the sensor is shown in Figure 6.

### 3.3. B-Scalar Meter

**Figure 7:**
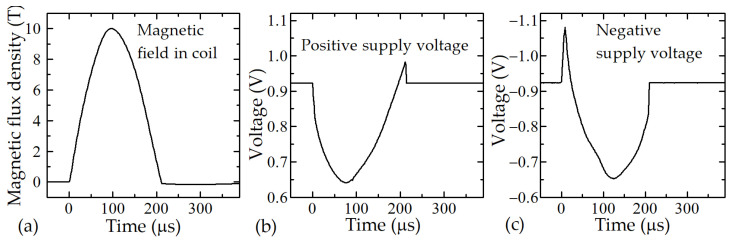
Magnetic field pulse generated in the center of a coil (**a**) and the response of the CMR-B-scalar sensor at positive (**b**) and negative (**c**) polarities of the supply voltage.

As discussed above, the absence of a memory effect of the sensors is necessary, but not sufficient for the requirements for measuring short magnetic field pulses. Although the chosen films at room and higher temperatures do not have the memory effect, there is still the problem with the induced electromotive force (EMF) in the connecting wires (the so-called loop effect), even when the wires are bifilarly twisted. In this case, the output signal (voltage) of the sensors contains two components: the voltage change caused by the sensor’s magnetoresistance (resistance decrease in applied magnetic field), and the parasitic voltage caused by the EMF induced in the loops of connecting wires. Some examples of the interference of these two signals are shown in Figure 7, which depicts the effect which occurred when a magnetic pulse with a duration of 200 μs was applied to the sensor in which the loop effect was not fully compensated.

**Figure 8:**
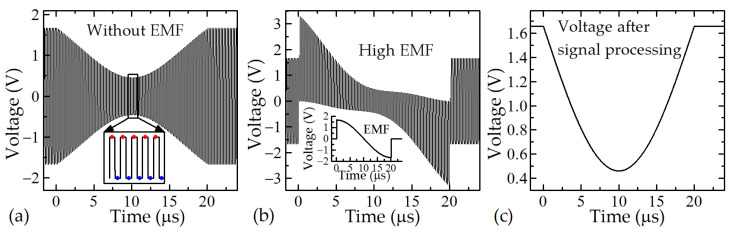
Voltage across the CMR sensor powered by pulsed voltage during application of pulsed magnetic field: (**a**) without the EMF; (**b**) with the EMF; (**c**) after signal processing. Inset: in (**a**)—points indicate time instants at which the ADC reads positive and negative voltage values; in (**b**)—EMF induced in the sensor and its wires.

In this study, the design of a novel magnetic field meter that allows the measurement of short magnetic pulses with compensation of parasitic EMF signal is proposed. This is achieved by using the bipolar-pulse supply of the CMR-B-scalar magnetic field sensor. At first, the LTspice simulation was performed. The results of the simulated voltage across the CMR sensor, supplied with a frequency of 5 MHz and exposed to a magnetic field pulse with a 20 μs duration, are shown in Figure 8. Figure 8a presents the result, when the EMF in the transmission line is absent. As one can see, due to the negative magnetoresistance of manganite film the positive and negative pulsed voltages are decreasing symmetrically during applied magnetic field pulse. In the second case (see Figure 8b), this change is affected by the electromotive force, which is proportional to the differential of the magnetic field in time. After the processing of these voltages according to Equation (2), the EMF is eliminated and the voltage change caused only by the change of resistance of the sensor in magnetic field is obtained (see Figure 8c).

*V*(*B*) = [(+*V*(*B*) + EMF) − (−*V*(*B*) + EMF)]/2. (2)

In this equation, +*V* and −*V* are the voltages of two adjacent pulses, when the sensor is supplied by positive and negative voltages, respectively (in the inset in Figure 8a, red and blue dots show the time instants, when the analog-to-digital converter (ADC) reads positive and negative voltage values).

**Figure 9:**
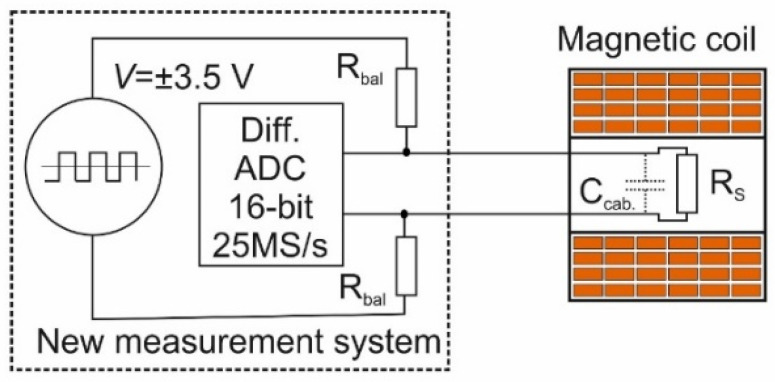
Block diagram of the new measurement system and magnetic coil used for testing and calibration of the sensor.

**Figure 10:**
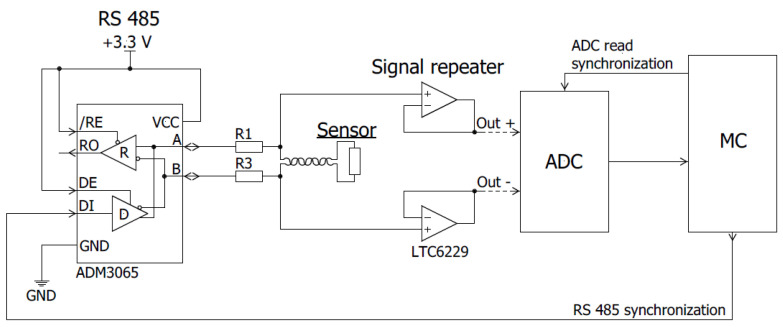
Circuit diagram of the sensor supply and digitalization of output signal.

A block diagram of the new proposed measurement system is shown in Figure 9. The CMR sensor (R_s_), connected with two ballast resistors (R_bal_) is supplied by a bipolar-pulse supply. The sensor is placed in the center of a coil of the magnetic field generator. Figure 10 shows a more detailed circuit of the power supply and recording system. It consists of a high-frequency bipolar-pulse power generator and high-frequency-differential analog-to-digital converter. A bipolar-pulse generator, based on the RS485 integral circuit, generates rectangular shape pulses with a frequency up to 12.5 MHz and is used as the main power source for the CMR sensor. The 16-Bit analog-to-digital converter (LTC2203) with a sampling rate up to 25 Msps is synchronized with the pulse generator and records the peak-to-peak negative and positive voltage across the sensor. A microprocessor (LPC54606J256BD100E) processes the output data of the ADC according Equation (2) (see inset in Figure 8a) and records it. As a result, the EMF is eliminated from the measured signal and only the useful voltage change caused by the magnetic field change is measured.

**Figure 11:**
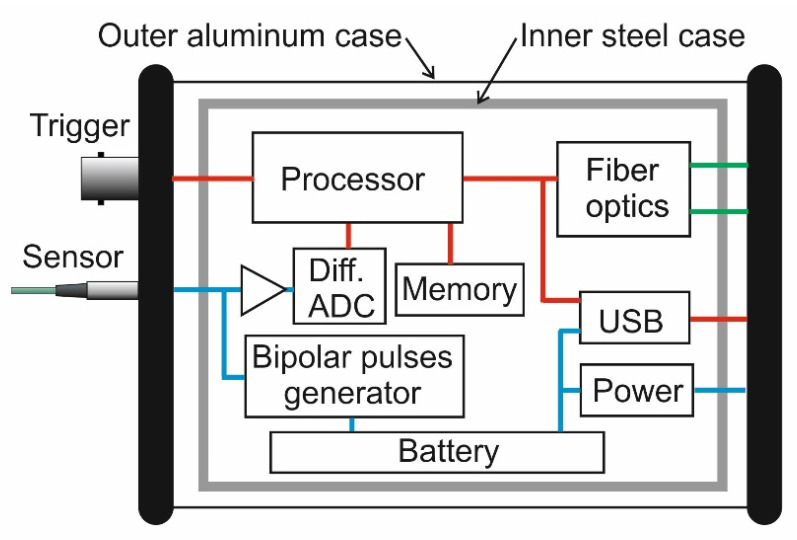
Main functional blocks of the fast B-scalar meter.

The proposed new magnetic field meter is an electronic device developed specially for measurements of short-pulsed magnetic fields. The casing of the meter consisting of an inner 4 mm thick steel box and an external 1.5 mm thick aluminum box is used to protect electronic circuits against EMI. The main functional components of the B-scalar meter are shown in Figure 11. The lithium-ion rechargeable battery (3.6 V) is used as a power supply for the electronic circuits. The internal power supply ensures that the device can be operated without being connected to any external power sources. The use of a high-resolution ADC allowed the signal conditioning circuits to be skipped, thus reducing the number of required analog components. After the processing of the measured signal, the voltage change is converted to the magnetic field and is stored. The sensor’s response is not a linear function of the magnetic flux density *B*. Moreover, it depends on the ambient temperature. Therefore, a calibration of sensors is necessary in order to assign a value of *B* to the magnetic-field-induced resistance change Δ*R* of the sensor (see Figure 3). The calibration tables of fabricated sensors (serial number is assigned to each sensor) are stored in the same measurement module.

The B-scalar meter is set up through its communication line only. Thus, it uses a protocol based on text commands. All operations of the B-scalar meter are performed only on request of the external controlling program (PC in-house software) and the trigger signal, which initiates the measurement. The software package developed for the short magnetic field measurement system consists of firmware installed in the B-scalar meter and control software for the PC. The firmware is responsible for the frequency in the generation of bipolar pulses, sampling the signal, storing the measured data, and sending it to the PC on demand. Thus, it controls the hardware of the measurement device and implements a communication protocol. The firmware supports basic functions of a digital oscilloscope, such as adjusting the frequency of generated pulses of the sensor supply source, and choosing the trigger source and the sensor number which is used. The measured signal from the measurement module is sent to the PC by fiber optic or through a USB cable. 

### 3.4. Experimental Results

**Figure 12:**
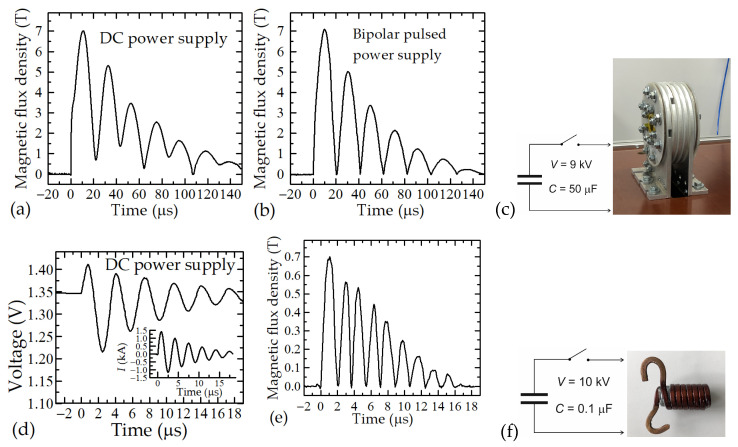
(**a**) magnetic field dynamics inside the Bitter coil when measurement was performed using the DC supply of the sensor; (**b**) magnetic field dynamics, when measurement was made by the new measurement system; (**c**) block diagram of magnetic field generation system with Bitter coil (see picture); (**d**) voltage change across the sensor positioned inside the two-layer coil when it was supplied by DC voltage; (**e**) magnetic field dynamic inside the two-layer coil when measurement was performed using the new measurement system; (**f**) block diagram of magnetic field generation system with two-layer coil (see picture).

The testing of the short magnetic field measurement system was performed using two types of pulsed magnetic field generators. The pulsed magnetic field was generated during the discharging of a capacitor bank through different coils. During the first experiment, a 50 µF capacitor bank charged up to 9 kV was discharged through a five-turn Bitter coil (*L* = 250 nH) by using a 100 kA spark-gap switch (see Figure 12c). During the second experiment, the 0.1 µF capacitor bank charged up to 10 kV was discharged through a two-layer coil (*L* = 1 µH) with seven turns in each layer (see Figure 12f). By discharging the capacitor bank through the coils, the magnetic field of the sine waveform with damped sinusoid amplitudes and frequencies of about 23 kHz and 263 kHz, respectively, was generated. As was mentioned before, the CMR-B-scalar sensor measures only the absolute value of the magnetic flux density *B*, and thus the obtained waveform of the magnetic field is unipolar. To illustrate the advantages of the new module, the magnetic field was measured using this new system and a system that was created earlier [18] in which the sensor was powered by a direct voltage (DC). The results of these investigations are shown in Figure 12. Figure 12a shows that during the first experiment, when the sensor was supplied with a DC voltage, the shape of the measured magnetic field was not semi-sinusoidal and was distorted. This is caused by the influence of the parasitic voltage created in the sensor’s wires due to the EMF effect. However, when measurements were made using a new system (see Figure 12b), the signal had the perfect form of a half-sine wave.

The advantages of the novel developed meter are mostly evident when measuring pulsed magnetic fields of several microseconds. Figure 12d shows the results of the voltage drop across the sensor during the applied short magnetic pulse. The inset of this figure shows the change in current flowing through the coil of the field generator. As it can be seen, the voltage across the sensor practically repeats the change of the current. This is caused by the generation of EMF in the sensor’s wires, which is much higher than the useful change in voltage caused by the magnetoresistance of the sensor. However, supplying the sensor with a bipolar voltage and processing this signal eliminates the induced parasitic EMF. The obtained final result is shown in Figure 12e. As can be seen, the voltage change corresponds only to the change of absolute value of the measured magnetic field.

## 4. Conclusions

A novel pulsed magnetic field measurement system, consisting of a CMR-B-scalar sensor based on thin manganite film and a fast measurement module, was developed for the measurement of the short-pulsed high-amplitude magnetic fields. The system ensures local field measurement as the active volume of the sensor is 400 × 50 × 0.40 μm^3^. 

It was found that nanostructured manganite LSMO film with Mn content increased above stoichiometric level results in the increase in the metal–insulator transition temperature *T*_m_ and an increase in the magnetoresistance values. Moreover, such films at temperatures higher than *T*_m_ do not exhibit magnetic memory effects and could be used for the measurement of short-pulsed magnetic fields at higher than room temperatures. For low (cryogenic) temperature applications, films doped with Co (LSMCO) are preferable due to minimized magnetic memory effect at these temperatures in comparison with the LSMO films. 

It was found that the parasitic EMF signal, induced in the wires of the sensor due to the high *dB*/*dt* during microsecond-duration magnetic pulses, has a great influence on the accuracy of the magnetic field measurement. It was demonstrated that the elimination of the EMF from the measured signal could be achieved by using a bipolar-pulse supply to drive the sensor. A bipolar-pulsed voltage generator with a frequency of up to 12.5 MHz, 16-Bit ADC with a sampling rate of 25 MHz, and a microprocessor allows the measurement of pulsed magnetic fields with pulse durations in the order of microseconds. 

To protect the electronic circuit against strong electromagnetic interference, a casing consisting of an internal thick steel box and an external thick aluminum box was used. The software package developed for the magnetic field measurement module and PC processes the measured signals and presents the obtained results in a user-friendly form. 

It was concluded that the created system can measure high-amplitude pulsed magnetic fields with a pulse duration in the order of microseconds, independent of the direction of the field. The system can be applied for measurements in very small volumes and can replace three-axis pick-up coil or B-dot sensors when the magnetic field direction is not known in advance. 

## Data Availability

Not applicable.

## References

[1] Abate D., Cavazzana R. (2022). Effective Area Measurements of Magnetic Pick-Up Coil Sensors for RFX-mod2. Sensors.

[2] Coupland J.H., Randle T.C., Watson M.J. (1981). A magnetic spectrometer with gradient field. IEEE Trans. Magn..

[3] Tumanski S. (2007). Indution coil sensors—A review. Meas. Sci. Technol..

[4] Wei S., Liao X., Zhang H., Pang J., Zhou Y. (2021). Recent Progress of Fluxgate Magnetic Sensors: Basic Research and Application. Sensors.

[5] Cho H.-S., Yang J.-H., Lee S.-Y., Lee J.-W., Lee J.-H. (2022). Wearable Fabric Loop Sensor Based on Magnetic–Field–Induced Conductivity for Simultaneous Detection of Cardiac Activity and Respiration Signals. Sensors.

[6] Marconato N. (2022). Design of Integrated Micro-Fluxgate Magnetic Sensors: Advantages and Challenges of Numerical Analyses. Sensors.

[7] Hosozawa A., Sekiguchi J., Asai T., Takahashi T. (2018). Application of a Hall sensor for pulsed magnetic field measurement in the FAT–CM FRC experiments. Rev. Sci. Instr..

[8] Kawahito S., Choi S.O., Ishida M., Nakamura T. (1994). Micromachined Hall elements for two-dimensional magnetic-field sensing. Sens. Actuators A Phys..

[9] Lenz J., Edelstein A.S. (2006). Magnetic sensors and their applications. IEEE Sens. J..

[10] Jogschies L., Klaas D., Kruppe R., Rittinger J., Taptimthong P., Wienecke A., Rissing L., Wurz M.C. (2015). Recent developments of magnetoresistive sensors for industrial applications. Sensors.

[11] Khan M.A., Sun J., Li B., Przybysz A., Kosel J. (2021). Magnetic sensors-A review and recent technologies. Eng. Res. Express.

[12] Murzin D., Mapps D.J., Levada K., Belyaev V., Omelyanchik A., Panina L., Rodionova V. (2020). Ultrasensitive Magnetic Field Sensors for Biomedical Applications. Sensors.

[13] Zheng C., Zhu K., de Freitas S.C., Chang J.-Y., Davies J.E., Eames P., Freitas P.P., Kazakova O., Kim C., Leung C.-W. (2019). Magnetoresistive sensor development roadmap (non-recording applications). IEEE Trans. Magn..

[14] Tang W., Lyu F., Wang D., Pan H. (2018). A new design of a single–device 3d Hall sensor: Cross–shaped 3D Hall sensor. Sensors.

[15] Li R., Zhang S., Luo S., Guo Z., Xu Y., Ouyang J., Song M., Zou Q., Xi L., Yang X. (2021). A spin–orbit torque device for sensing three-dimensional magnetic fields. Nat. Electron..

[16] Shiogai J., Fujiwara K., Nojima T., Tsukazaki A. (2021). Three-dimensional sensing of the magnetic-field vector by a compact planar-type Hall device. Commun. Mater..

[17] Alfadhel A., Carreno A.A.A., Foulds I.G., Kosel J. (2013). Three-Axis Magnetic Field Induction Sensor Realized on Buckled Cantilever Plate. IEEE Trans. Magn..

[18] Stankevič T., Medišauskas L., Stankevič V., Balevičius S., Žurauskiene N., Liebfried O., Schneider M. (2014). Pulsed magnetic field measurement system based on colossal magnetoresistance-B-scalar sensors for railgun investigation. Rev. Sci. Instrum..

[19] Haran T.L., Hoffman R.B., Lane S.E. (2013). Diagnostic capabilities for electromagnetic railguns. IEEE Trans. Plasma Sci..

[20] Balevičius S., Žurauskiene N., Stankevič V., Keršulis S., Plaušinaitiene V., Abrutis A., Zherlitsyn S., Herrmannsdörfer T., Wosnitza J., Wolff–Fabris F. (2012). Nanostructured thin manganite films in megagauss magnetic field. Appl. Phys. Lett..

[21] Žurauskiene N., Pavilonis D., Klimantavicius J., Balevičius S., Stankevič V., Vasiliauskas R., Plaušinaitiene V., Abrutis A., Skapas M., Juškenas R. (2017). Magnetoresistance relaxation anisotropy of nanostructured La-Sr(Ca)-Mn-O films induced by high-pulsed magnetic fields. IEEE Trans. Plasma Sci..

[22] Portugall O., Solane P.Y., Plochocka P., Maude D.K., Nicholas R.J. (2013). Beyond 100 Tesla: Scientific experiments using single-turn coils. Comptes Rendus Phys..

[23] Portugall O., Puhlmann N., Muller H.U., Barczewski M., Stolpe I., von Ortenberg M. (1999). Megagauss magnetic field generation in single-turn coils: New frontiers for scientific experiments. J. Phys. D Appl. Phys..

[24] Novac B.M., Smith I.R., Rankin D.F., Pu Z., Hubbard M. Electromagnetic flux-compression: Experimentation. Proceedings of the 14th IEEE International Pulsed Power Conference.

[25] Novac B.M., Hook N.D., Smith I.R. Magnetic flux-compression driven by exploding single-turn coils. Proceedings of the IEEE International Power Modulator and High Voltage Conference.

[26] Bellmann J., Lueg-Althoff J., Schulze S., Gies S., Beyer E., Tekkaya A.E. (2016). Measurement and analysis technologies for magnetic pulse welding: Established methods and new strategies. Adv. Manuf..

[27] Broeckhove J., Willemsens L., Faes K., DeWaele W. (2011). Magnetic pulse welding. Int. J. Sustain. Constr. Des..

[28] Sirena M., Steren L.B., Guimpel J. (2001). Magnetic relaxation in bulk and film manganite compounds. Phys. Rev. B.

[29] Zurauskiene N., Balevicius S., Pavilonis D., Stankevic V., Kersulis S., Novickij J. (2013). Magnetoresistance relaxation in thin La-Sr-Mn-O films exposed to high-pulsed magnetic fields. IEEE Trans. Plasma Sci..

[30] Zurauskiene N., Rudokas V., Balevicius S., Kersulis S., Stankevic V., Vasiliauskas R., Plausinaitiene V., Vagner M., Lukose R., Skapas M. (2017). Nanostructured La–Sr–Mn–Co–O films for room-temperature pulsed magnetic field sensors. IEEE Trans. Magn..

[31] Zurauskiene N., Rudokas V., Kersulis S., Stankevic V., Pavilonis D., Plausinaitiene V., Vagner M., Balevicius S. (2021). Magnetoresistance and its relaxation of nanostructured La–Sr–Mn–Co–O films: Application for low temperature magnetic sensors. J. Magn. Magn. Mater..

[32] Zurauskiene N., Stankevic V., Kersulis S., Klimantavicius J., Simkevicius C., Plausinaitiene V., Vagner M., Balevicius S. (2019). Increase of operating temperature of magnetic field sensors based on La–Sr–Mn–O films with Mn excess. IEEE Trans. Plasma Sci..

[33] Balevicius S., Zurauskiene Z., Stankevic V., Stankevic T., Novickij J., Schneider M. (2013). High-Frequency CMR–B–Scalar Sensor for Pulsed Magnetic Field Measurement. IEEE Trans. Plasma Sci..

[34] Mironov O.A., Zherlitsyn S., Uhlarz M., Skoursli Y., Palewski T., Wosnitza J. (2010). Micro-miniature Hall probes for applications at pulsed magnetic fields up to 87 Tesla. J. Low Temp. Phys..

[35] Imamura H., Uchida K., Ohmichi E., Osada T. (2006). Magnetotransport measurements of low dimensional conductors under pulsed ultra-high magnetic fields. J. Phys. Conf. Ser..

[36] Zurauskiene N., Balevicius S., Stankevic V., Kersulis S., Klimantavicius J., Plausinaitiene V., Kubilius V., Skapas M., Juskenas R., Navickas R. (2018). Magnetoresistive properties of thin nanostructured manganite films grown by metalorganic chemical vapour deposition onto glass-ceramics substrates. J. Mater. Sci..

[37] Abrutis A., Plausinaitiene V., Kubilius V., Teiserskis A., Saltyte Z., Butkute R., Senateur J.P. (2002). Magnetoresistant La1yxSrxMnO3 films by pulsed injection metal organic chemical vapor deposition: Effect of deposition conditions, substrate material and film thickness. Thin Solid Films.

[38] Lukose R., Plausinaitiene V., Vagner M., Zurauskiene N., Kersulis S., Kubilius V., Naujalis E. (2019). Relation between thickness, crystallite size and magnetoresistance of nanostructured La_1−x_Sr_x_MnyO_3±δ_ films for magnetic field sensors. Beilstein J. Nanotechnol..

[39] Novickij J., Balevicius S., Zurauskiene N., Kacianauskas R., Stankevic V., Simkevicius C., Kersulis S., Bartkevicius S. (2010). Vilnius high magnetic field centre facilities. J. Low Temp. Phys..

